# Supplementary material for: The Evolution and Characterization of the RNA Interference Pathways in Lophotrochozoa
Source: Genome Biol Evol. 2024 May 7;16(5):evae098. doi: 10.1093/gbe/evae098 (PMC11114477; doi:10.1093/gbe/evae098)
Supplement: evae098_Supplementary_Data [file evae098_supplementary_data.zip › Supplementary_materials.zip!/selected_miRNAs/DaRe_selected_miRNas/DaRe_NC_007128.7_30263.pdf]

Provisional ID	:	NC_007128.7_30263
Score total	:	98.5
Score for star read(s)	:	3.9
Score for read counts	:	90.4
Score for mfe	:	3.3
Score for randfold	:	1.6
Score for cons. seed	:	-0.6
Total read count	:	189
Mature read count	:	151
Loop read count	:	0
Star read count	:	38

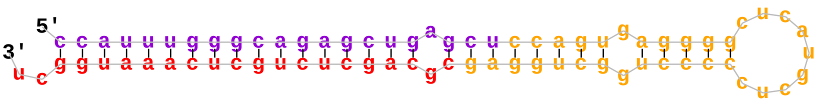

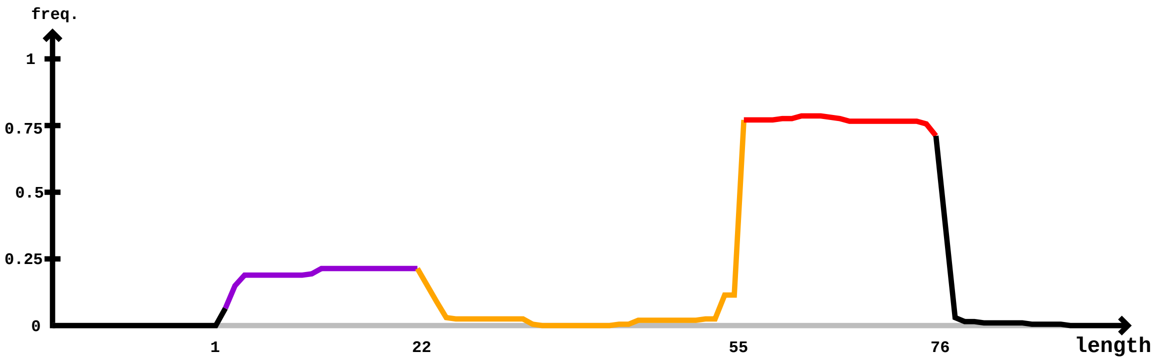

Star Mature

5'- ggugcucacugcagagccauuugggcagagcugagcuccagugaggggcucaugcuccccccuggcuggagcgcagcucugcucaaauggcucugcagcgagcagugucuc -3'	obs		
ggugcucacugcagagccauuugggcagagcugagcuccagugaggggcucaugcuccccccuggcuggagcgcagcucugcucaaauggcucugcagcgagcagugucuc	exp		
(.((((.(((((((((((((((((((((((((.((((((((.(((((.........))))).)))))))).))))))))))))))))))))))))).))))).).....	reads	mm	sample
................ccauuugggcagagcugagcu..	3	0	Gu2
.................cauuugggcagagcugagcu..	1	0	Gu2
.................cauuugggcagagcugagcuc...	1	0	Gu2
.................cauuugggcagagcugagcucU..	1	1	Gu2
.................cauuugggcGgagcugagcucc..	1	1	Gu2
....ccuggcuggagcgcagcucug..............................	1	0	Gu2
....ccuggcuggagcgcagcucugc.............................	2	0	Gu2
....agcgcagcucugcucaaauggA....................	1	1	Gu2
....agcgcagcucugcucaaauggG....................	1	1	Gu2
....agcgcagcucugcucaaauggcu...................	1	0	Gu2
....cgcagcucugcucaaauggc....................	2	0	Gu2
....cgcagcucugcucaaauggcC...................	13	1	Gu2
....cgcagcucugcucaaauggcu...................	6	0	Gu2
....cgcagcucugcucaaauggcA...................	1	1	Gu2
....cgcagcucugcucaaauggcuA..................	1	1	Gu2
.................cauuugggcagagcugagcuc...	1	0	Ov3
....ggGgcgcagcucugcucaaauggcu...................	1	1	Ov3
....cgcagcucugcucaaaugg.....................	1	0	Ov3
....cgcagcucugcucaaGuggcu...................	1	1	Ov3
....cgcagcucugcucaaauggcA...................	2	1	Ov3
....cgcagcucugcucaaauggcC...................	5	1	Ov3
....cgcagcucugcucaaauggcu...................	4	0	Ov3
................ccauuugggcagagcugagcu..	4	0	Gu1
.................cauuugggcagagcugagcu..	1	0	Gu1
.................cauuugggcagagcugagcuc...	5	0	Gu1
.................cauuugggcagagcugagcucc..	1	0	Gu1
.................cauuugggcagagcugagcucA..	1	1	Gu1
.................cauuugggcagagcugagcucca...	1	0	Gu1
..................auuugggcagagcugagcuc...	1	0	Gu1
..................auuugggcagagcugagcucc..	2	0	Gu1
..........................agagcugagcuccagugagggC...	1	1	Gu1

Star **Mature**

```
ggugcucacugcagagccauuugggcagagcugagcuccagugaggggcucaugcucccccuggcuggagcgcagcucugcucaaauggcucugcagcgagcagugucuc
..........................agagcugagcuccagugagggg..............................................................    1    0    Gu1
.........................................................ccccuggcuggagcgcagcucu...............................    1    0    Gu1
....................................................................agcgcagcucugcucaaaugg.....................    1    0    Gu1
....................................................................agcgcagcucugcucaaauggc....................    2    0    Gu1
....................................................................Cgcgcagcucugcucaaauggcu...................    1    1    Gu1
....................................................................agcgcagcucugcucaaauggcC...................    7    1    Gu1
....................................................................agcgcagcucugcucaaauggcu...................    1    0    Gu1
......................................................................cgcagcucugcucaGauggcu...................    3    1    Gu1
......................................................................cgcagcucugcucaaauggcC...................    17   1    Gu1
......................................................................cgcagcucugcucaaauggcu...................    26   0    Gu1
......................................................................cgcagcucugcucaaauggcA...................    1    1    Gu1
......................................................................cgcagcucugcucaaauggcG...................    1    1    Gu1
......................................................................cgcGgcucugcucaaauggcu...................    3    1    Gu1
......................................................................cgcagcucugcucaaauggcuc..................    1    0    Gu1
......................................................................cgcagcucugcucaaauggcuU..................    5    1    Gu1
......................................................................cgcagcucugcucaaauggcCc..................    1    1    Gu1
......................................................................cgcagcucugcucaaauggcCcu.................    1    1    Gu1
......................................................................cgcagcucugcucaaauggcucC.................    1    1    Gu1

.................cauuugggcagagcugagcucc.......................................................................    1    0    Ov1
..................auuugggcagagcugagcucc.......................................................................    1    0    Ov1
....................................................................agcgcagcucugcucaaauggcC...................    1    1    Ov1
......................................................................cgcagcucugcucaGauggc....................    1    1    Ov1
......................................................................cgcagcucugcucaaauggcC...................    6    1    Ov1
......................................................................cgcagcucugcucaaauggcu...................    2    0    Ov1
......................................................................cgcagcucugcucaaauggcCc..................    1    1    Ov1
......................................................................cgcagcucugcucaaauggcucC.................    1    1    Ov1
............................................................................ucugcucaaauggcucugcagcgagcaC......    1    1    Ov1

................ccauuugggcagagcugagcu.........................................................................    5    0    Gu3
................ccauuugggcagagcugagcuc........................................................................    1    0    Gu3
.................cauuugggcagagcugagcucc.......................................................................    1    0    Gu3
..................auuugggcagagcugagcuc........................................................................    1    0    Gu3
..................auuugggcagagcugagcucc.......................................................................    3    0    Gu3
.........................cagagcugagcuccagugagggU..............................................................    1    1    Gu3
..........................agagcugagcuccagugagggC..............................................................    1    1    Gu3
..........................agagcugagcuccagugaggggc.............................................................    1    0    Gu3
....................................................................agcgcagcucugcucaaauggc....................    1    0    Gu3
....................................................................agcgcagcucugcucaaauggA.....................    1    1    Gu3
......................................................................cgcagcucugcucaaauggcA...................    2    1    Gu3
......................................................................cgcagcucugcucaaauggcu...................    7    0    Gu3
......................................................................cgcagcucugcucaaauggcC...................    6    1    Gu3
......................................................................cgcagcucugcucaGauggcu...................    1    1    Gu3
......................................................................cgcagcucugcucaaauggcuU..................    2    1    Gu3
......................................................................cgcagcucugcucaaauggcCc..................    1    1    Gu3

.................cauuugggcagagcugagcuc........................................................................    1    0    Ov2
......................................................................cgcagcucugcucaaauggcC...................    4    1    Ov2
......................................................................cgcagcucugcucaaauggcu...................    2    0    Ov2
..........................................................................gcucugcucaGauggcucugc...............    1    1    Ov2
............................................................................ucuCcucaaauggcucugcagcga..........    1    1    Ov2
```
